# Retracted: System Accuracy Evaluation of the GlucoRx Nexus Voice TD-4280 Blood Glucose Monitoring System

**DOI:** 10.1155/2015/496928

**Published:** 2015-06-17

**Authors:** Disease Markers

The paper titled “System Accuracy Evaluation of the GlucoRx Nexus Voice TD-4280 Blood Glucose Monitoring System” [1], published in Disease Markers, has been retracted upon the authors' request, as the routine hospital laboratory evaluation protocol used varied from recommended best practice study design. Although this protocol established the fact that GlucoRx Nexus Voice system was clinically accurate, it could not be used to make valid analytical accuracy statements relating to ISO 15197 standards. GlucoRx Nexus meters are now the formulary choice in the area.
